# Calculating Evolutionary Dynamics in Structured Populations

**DOI:** 10.1371/journal.pcbi.1000615

**Published:** 2009-12-18

**Authors:** Charles G. Nathanson, Corina E. Tarnita, Martin A. Nowak

**Affiliations:** 1 Department of Economics, Harvard University, Cambridge, Massachusetts, United States of America; 2 Program for Evolutionary Dynamics, Department of Mathematics, Department of Organismic and Evolutionary Biology, Harvard University, Cambridge, Massachusetts, United States of America; University of Washington, United States of America

## Abstract

Evolution is shaping the world around us. At the core of every evolutionary process is a population of reproducing individuals. The outcome of an evolutionary process depends on population structure. Here we provide a general formula for calculating evolutionary dynamics in a wide class of structured populations. This class includes the recently introduced “games in phenotype space” and “evolutionary set theory.” There can be local interactions for determining the relative fitness of individuals, but we require global updating, which means all individuals compete uniformly for reproduction. We study the competition of two strategies in the context of an evolutionary game and determine which strategy is favored in the limit of weak selection. We derive an intuitive formula for the structure coefficient, σ, and provide a method for efficient numerical calculation.

## Author Summary

At the center of any evolutionary process is a population of reproducing individuals. The structure of this population can greatly affect the outcome of evolution. If the fitness of an individual is determined by its interactions with others, then we are in the world of evolutionary game theory. The population structure specifies who interacts with whom. We derive a simple formula that holds for a wide class of such evolutionary processes. This formula provides an efficient computational method for studying evolutionary dynamics in structured populations.

## Introduction

Constant selection implies that the fitness of individuals does not depend on the composition of the population. In general, however, the success of individuals is affected by what others are doing. Then we are in the realm of game theory [1]–[3] or evolutionary game theory [4]–[8]. The latter is the study of frequency dependent selection; the fitness of individuals is typically assumed to be a linear function of the frequencies of strategies (or phenotypes) in the population. The population is trying to adapt on a dynamic fitness landscape; the changes in the fitness landscape are caused by the population that moves over it [9]. There is also a close relationship between evolutionary game theory and ecology [10]: the success of a species in an ecosystem depends on its own abundance and the abundance of other species.

The classical approach to evolutionary game dynamics is based on deterministic differential equations describing infinitely large, well-mixed populations [6],[11]. In a well-mixed population any two individuals interact equally likely. Some recent approaches consider stochastic evolutionary dynamics in populations of finite size [12],[13]. Evolutionary game dynamics are also affected by population structure [14]–[22]. For example, a well-mixed population typically opposes evolution of cooperation, while a structured population can promote it. There is also a long standing tradition of studying spatial models in ecology [23]–[25], population genetics [26],[27] and inclusive fitness theory [28]–[30].

Evolutionary graph theory is an extension of spatial games, which are normally studied on regular lattices, to general graphs [31]–[34]. The graph determines who meets whom and reflects physical structure or social networks. The payoff of individuals is derived from local interactions with their neighbors on the graph. Moreover, individuals compete locally with their neighbors for reproduction. These two processes can also be described by separate graphs [35].

‘Games in phenotype space’ [36] represent another type of spatial model for evolutionary dynamics, which is motivated by the idea of tag based cooperation [37]–[39]. In addition to behavioral strategies, individuals express other phenotypic features which serve as markers of identification. In one version of the model, individuals interact only with those who carry the same phenotypic marker. This approach can lead to a clustering in phenotype space, which can promote evolution of cooperation [36].

‘Evolutionary set theory’ represents another type of spatial model [40]. Each individual can belong to several sets. At a particular time, some sets have many members, while others are empty. Individuals interact with others in the same set and thereby derive a payoff. Individuals update their set memberships and strategies by global comparison with others. Successful strategies spawn imitators, and successful sets attract more members. Therefore, the population structure is described by an ever changing, dynamical graph. Evolutionary dynamics in set structured populations can favor cooperators over defectors.

In all three frameworks – evolutionary graph theory, games in phenotype space and evolutionary set theory – the fitness of individuals is a consequence of local interactions. In evolutionary graph theory there is also a local update rule: individuals learn from their neighbors on the graph or compete with nearby individuals for placing offspring. For evolutionary set theory, however, [40] assumes global updating: individuals can learn from all others in the population and adopt their strategies and set memberships. Global updating is also a feature of the model for games in phenotype space [36]. The approach that is presented in this paper requires global updating. Therefore, our result holds for evolutionary set theory and for games in phenotype space, but does not apply to evolutionary graph theory.

## Results

Consider a game between two strategies, 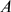 and 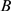. If two 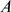 players interact, both get payoff 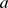; if 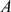 interacts with 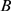, then 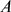 gets 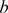 and 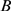 gets 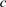; if two 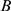 players interact, both get 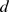. These interactions are represented by the payoff matrix
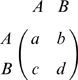
(1)We consider a population of finite size 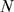. Each individual uses either strategy 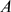 or 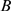. In the framework that we investigate here, the population structure specifies how people interact to derive their payoff. It could be that some individuals interact while others do not, or that some individuals interact stronger or more frequently than others. For example, in evolutionary set theory individuals interact with others who are in the same set and two individuals interact as many times as they have sets in common; in games in phenotype space, individuals interact with others who share the same phenotype.

Based on these interactions, individuals derive a cumulative payoff, 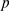. The fitness of an individual is given by 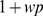 where the parameter 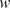 characterizes the intensity of selection. In this paper we consider the limit of weak selection, 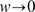.

Reproduction is proportional to fitness but subject to mutation. With probability 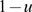 the offspring adopts the strategy of the parent. With probability 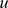 a random strategy is chosen (which is either 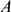 or 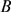).

A state of the population contains all information that can affect the payoffs of players. It assigns to each player a strategy (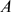 or 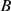) and a ‘location’ (in space, phenotype space etc). Thus, one can think of a state as a binary vector which specifies the strategy of each individual, together with a real 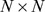 matrix whose 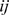-th entry specifies the weight of the interaction of individual 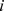 with 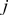. For example, in evolutionary set theory, the 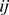-th entry of this matrix gives the number of sets 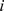 and 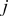 have in common [40]. Note that this matrix is not necessarily symmetric: the weight of 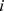
's interaction with 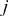 might be different from the weight of 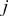
's interaction with 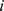. In this paper, whenever we refer to the number of interactions between individuals, we always count them with their weights or multiplicities.

For our proof we assume a finite state space and we study the Markov process defined by gameplay together with the update rule on this state space. The Markov process has a unique stationary distribution defined over all states.

It is shown in [41] that for weak selection, the condition that 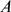 is more abundant than 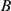 in the stationary distribution of the mutation-selection process described above can be written as
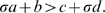
(2)Therefore, the crucial condition specifying which strategy is more abundant is a linear inequality in the payoff values, 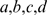. The structure coefficient, 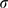, can depend on the population structure, the update rule, the population size and the mutation rate, but not on the payoff values, 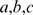 and 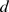. This ‘structural dominance’ condition (2) holds for a wide variety of population structures and update rules, including games in well mixed populations [12],[13], games on graphs [32]–[34], games in phenotype space [36] and games in set structured populations [40].

For a large well-mixed population we obtain 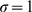. Therefore, the standard risk-dominance type condition, 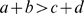, specifies if 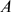 is more abundant than 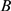. Spatial structure leads to 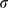 values that are greater than 1. The larger 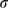 the greater is the deviation from the well mixed population. For very large 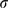 strategy 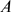 is more abundant than 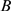 if 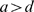. Therefore, spatial structure promotes Pareto efficiency over risk dominance [41]. If a spatial model generates 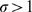 then it is a mechanism for the evolution of cooperation [42].

Here we derive a formula for 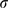 that holds for all processes satisfying two conditions:

- *global updating*, which means individuals compete uniformly with all others for reproduction and
- *constant birth or death rate* which means the payoff from the game can affect either the birth rate or the death rate but not both.

These assumptions are fulfilled, for example, by games in phenotype space [36] and by games on sets [40]. They do not hold, however, for games on graphs [32]. The first assumption is necessary because our calculation requires that the update rule depends only on fitness, and not on locality. Local update rules are less well-behaved and can even lead to negative values of 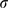. The second assumption insures that the change in the frequency of players is due only to a change in selection. Without this second assumption the conditions would be more complicated.

**Figure 1:**
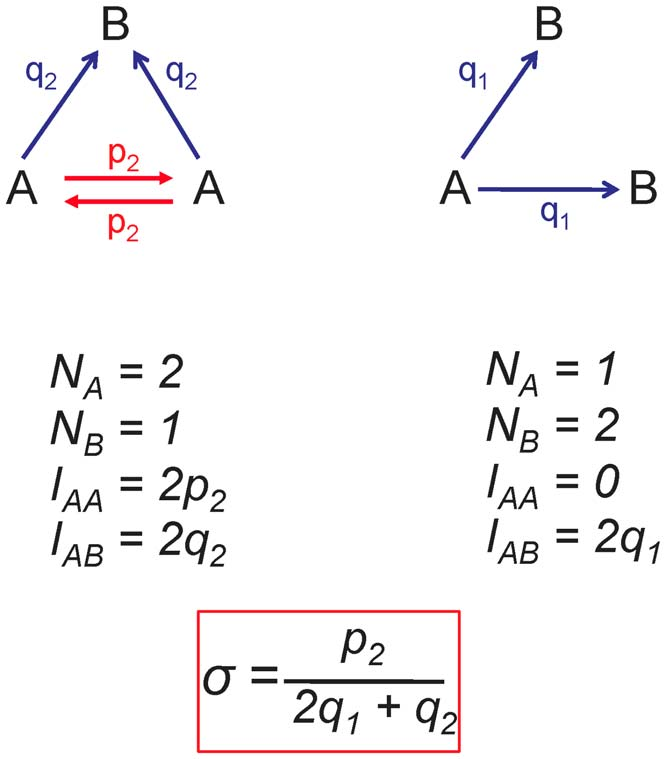
Calculation of 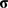 for a very simple example with population size 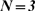. Suppose there is a ‘spatial’ process which has two mixed states. These two states must have the same frequency in the stationary distribution at neutrality, because the process cannot introduce asymmetries between 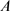 and 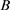 at neutrality. Each mixed state can be described by a weighted, directed graph: in a state with 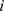 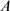 players, let 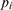 be the probability that an 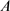 plays with another 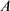 and let 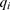 be the probability that an 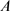 plays with a 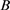. These probabilities are enough since for the calculation of 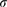 we only need the 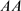 edges and the 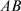 edges. Note also that the pure states, all-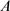 and all-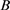, do not contribute to the calculation. We obtain 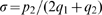.

For each state of the system, let 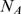 be the number of individuals using strategy 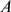; the number of individuals using strategy 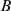 is 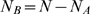. Furthermore, let 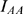 denote the total number of encounters that 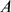 individuals have with other 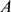 individuals. Note that every 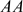 pair is counted twice because each 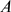 individual in the pair has an encounter with another 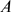 individual. As specified before, whenever we say ‘number of interactions’ we count the interactions together with their weights (if such weights occur in the model). Let 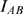 denote the total number of interactions that an 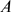 individual has with 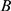 individuals. Our main result is that the structure coefficient, 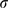, can be written as
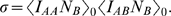
(3)The notation 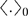 means that the quantity is averaged over all states of the stochastic process under neutral drift, 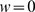; each term of the average is weighted by the frequency of the corresponding state in the stationary distribution. Intuitively, 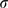 captures how much more likely it is, on average, for an individual to play with his own kind rather than with the other kind. An illustration of this formula is shown in Figure 1.

This formula suggests a simple numerical algorithm for calculating the 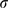-factor for any spatial process with global updating. We let the process run for a very long time assuming that all individuals have the same fitness. Thus, we simulate mutation and neutral drift on a spatial structure. For each state we evaluate 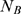, 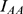, and 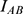. We add up all 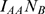 terms to get the numerator in eq (3). We add up all 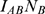 terms to get the denominator. The resulting 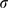 can be used for any game given by the payoff matrix (1) to determine if strategy 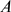 is more frequent than strategy 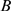 in the limit of weak selection.

The rigorous proof of eq (3) is given in Appendix A; here we provide an intuition for it. For symmetry reasons, at neutrality, we have the following identities 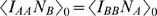 and 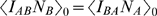. Using these symmetries together with our formula (3), we rewrite condition (2) as
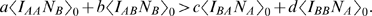
(4)Denoting by 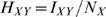 the average number of interactions of 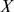 individuals with 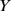 individuals, we can further rewrite eq. (4) as
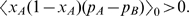
(5)Here 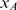 is the frequency of 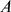 individuals, 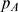 is the average payoff of an 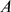-individual and 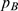 is the average payoff of a 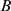-individual. These are 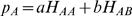 and 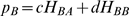.

A standard replicator equation for deterministic evolutionary game dynamics of two strategies in a well-mixed population can be written as 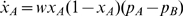 where 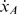 is the time derivative of the change due to selection and 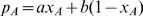 and 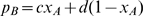 denote the average payoffs for 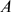 and 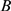 if the frequency of 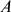 is 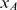. This equation describes how selection alone changes the frequency of strategy 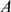 over time. Hence, the condition that strategy 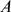 is favored by selection is 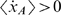 where the average is now taken over all states of the mutation-selection process, in the presence of game (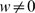). In the limit of weak selection, one can write the first-order Taylor expansion of this inequality to obtain 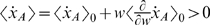. Since at neutrality the average change in the frequency of 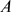 is zero, our condition for strategy 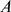 to be favored over strategy 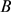 becomes 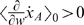 which is precisely inequality (5). Therefore inequality (5) has a very intuitive interpretation.

### Evolution of cooperation

As a particular game we can study the evolution of cooperation. Consider the simplified Prisoner's Dilemma payoff matrix:
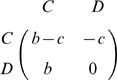
(6)This means cooperators, 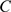, pay a cost 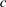 for others to receive a benefit, 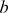. Defectors, 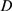, pay no cost and distribute no benefits. The game is a Prisoner's Dilemma if 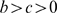.

As shown in [41], if we use equation (2) we can always write the critical benefit-to-cost ratio as
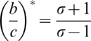
(7)provided 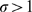. If the benefit-to-cost ratio exceeds this critical value, then cooperators are more abundant than defectors in the mutation-selection equilibrium of the stochastic process for weak selection. A higher 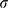 corresponds to a lower benefit-to-cost ratio and is thus better for the evolution of cooperation.

From eqs (3) and (7) we can write
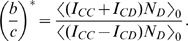
(8)This formula is very useful for finding the critical benefit-to-cost ratio numerically. Moreover, we can rewrite the critical benefit-to-cost ratio in terms of average number of interactions rather than total number of interactions as
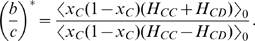
(9)These equations provide intuitive formulations of the critical benefit-to-cost ratio for processes with global updating.

### Computational example: Evolutionary dynamics on sets

Our new formula for 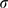 (eq. 3) gives a simple numerical algorithm for calculating this quantity in any spatial process with global updating and constant birth or death rate. We simulate this process under neutral drift for many generations. For each state we evaluate 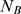, 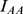, and 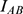. We add up all 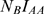 products to get the numerator in eq (3), and then we add up all 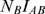 products to get the denominator. The resulting 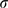 can be used for any game given by the payoff matrix (1) to determine if strategy 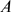 is more frequent than strategy 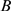 in the limit of weak selection.

**Figure 2:**
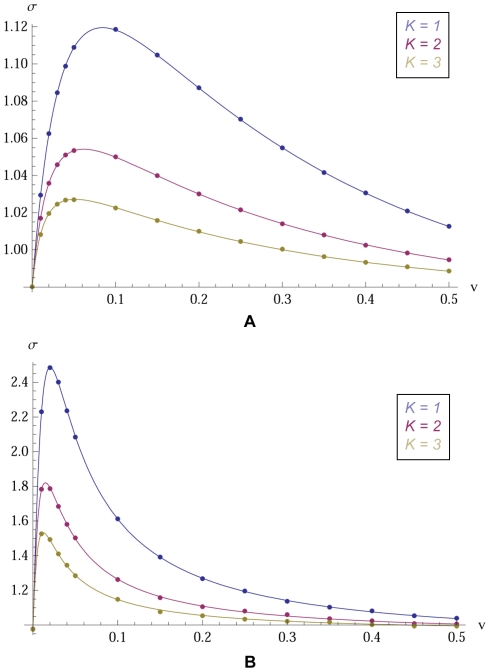
Agreement of simulations with analytic results. We test our simulation procedure against the analytic results of the set model of [40]. Parameters used are 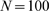 and 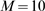. 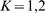 or 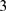 is the number of sets an individual is in, 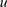 is the strategy mutation, and 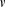 is the set mutation. We run simulations for 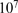 generations. We use a low strategy mutation 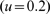 in (A) and a high strategy mutation 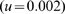 in (B).

**Figure 3:**
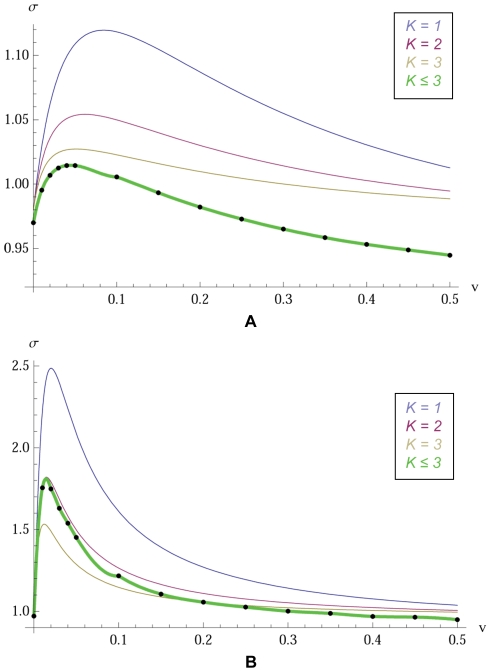
Simulated results for model with variable number of set memberships. An individual can be in 1, 2, or 3 sets; when he mutates set membership, the number of sets he joins is drawn with uniform probability. Parameter values are 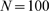, 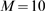; 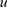 is the strategy mutation rate and 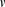 is the set mutation rate. We run the simulation using the method of eq. (3) for 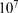 generations. Dots indicate simulated results, which are interpolated with a smooth curve. This variable set membership model has not yet been solved analytically. (A) The interpolated curve for small strategy mutation 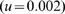 compared to the analytical result for 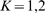 or 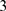. (B) The interpolated curve for high strategy mutation rate 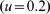 compared to the analytical result for 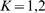 or 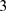.

In this section we use the simple numerical algorithm suggested by our formula (3) to find 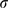 for evolutionary dynamics on sets [40]. In that paper, the authors compute an exact analytic formula for 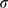 that depends on the parameters of their model. We compare our simulated estimates for 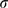 with their theoretical values and find perfect agreement (Figure 2). Furthermore, we use our computational method to calculate 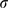 in an extension of the original model. An analytic solution for this extended model has not yet been found. Thus our simulated estimates constitute the first “solution” of this extended model (Figure 3).

The original set-structured model describes a population of 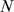 individuals distributed over 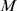 sets. Individuals interact with others who belong to the same set. Two individuals interact as many times as they have sets in common, and these interactions lead to payoffs from a game as described in general in Section 2. Reproductive updating follows a Wright-Fisher process, where 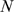 individuals are selected with replacement to seed the next generation. The more fit an individual, the more likely it is to be chosen as a parent. An offspring adopts the parent's strategy with probability 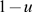, as described in Section 2. The offspring adopts the parent's set memberships, but this inheritance is also subject to mutation; with probability 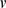, an offspring adopts a random list of set memberships. This updating process can be thought of as imitation-based dynamics where both strategies and set memberships are subject to selection [40].

To obtain exact analytical calculations, it is assumed that each individual belongs to exactly 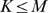 sets. In Figure 2, we pick values for 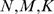, and 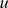 and plot 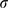 as a function of the set mutation rate, 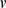. The continuous curves are based on the analytic formula for 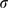 derived in [40]. The new numerical algorithm generates the data points. There is perfect agreement between these two methods.

In Figure 3, we consider a variant of this model. Instead of belonging to exactly 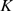 sets, individuals now belong to *at most* 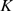 sets. With probability 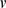, an offspring adopts a random list of *at most* 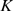 memberships, the length of which is uniformly random. So far there exists no analytical solution for this model but we can use eq. (3) to compute 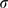 numerically. We interpolate the numerical results with smooth curves. We observe that for low mutation, Fig. 3(A), the case 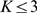 gives a 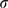 which is smaller than the 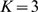 case. Hence, for low mutation, allowing people to be in *at most* 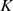 sets turns out to be worse for cooperation than restricting them to be in *exactly* 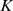 sets. However, for high strategy mutation, Fig. 3(B), the 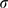 for 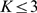 is greater than the one for 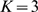. Hence, for high strategy mutation, allowing individuals to be in *at most* 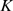 sets seems to be better for cooperation than restricting them to be in *exactly* 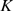 sets. This suggests that there exists an intermediate strategy mutation rate where the two cases are similar.

## Discussion

It has been shown that evolutionary dynamics in a structured population can be described by a single parameter, 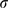, if we are merely interested in the question, which of the two competing strategies, 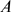 or 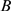, is more abundant in the limit of weak selection [41]. Payoff matrix (1) describes the interaction between the two strategies 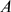 and 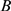 and the inequality 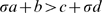 specifies that 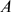 is more abundant than 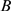 in the mutation-selection equilibrium. In general the parameter 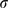 can depend on the population structure (which specifies who interacts with whom for accumulating payoff and for evolutionary updating), the population size and the mutation rates; but it does not depend on the entries of the payoff matrix. The 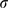 parameter has been explicitly calculated for a number of models including games on graphs, games in phenotype space, games in set structured populations and a simple model of multi-level selection [42].

Here we provide a general formula for the 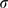 factor, which holds for the case of global updating. Global updating means that all members of the population compete globally (as opposed to locally) for reproduction. For example, global updating arises in the following way: one individual reproduces and another random individual dies (in order to maintain constant population size); the offspring of the first individual might inherit (up to mutation) the strategy and the ‘location’ of the parent. Global updating is a feature of models for games in phenotype space [36] and for games on sets [40].

Our main result, eq (3), provides both an intuitive description of what the 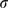 factor is and an efficient way for numerical computation.

## Materials and Methods

Here we give the proof of equation (3). It is based on the following three claims which we prove in the next subsection:

### 

#### Claim 1

First, we show that for structures and update rules with either constant death rate or constant birth rate the condition
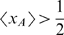
(10)for strategy 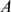 to be favored over strategy 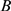 is equivalent to
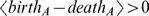
(11)where 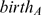 and 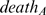 are the total birth and death rates of 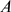 players and 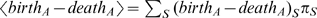 is the change due to selection averaged over all states of the system, weighted by the probability 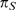 that the system is in each state. The change due to selection in the frequency of 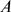 in each state is the difference between the number of 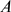
's that are born and the number of 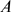
's that die.

#### Claim 2

We show that for global updating, condition (11) is equivalent to
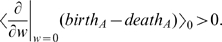
(12)Here 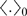 denotes the average over the stationary distribution in the neutral process, 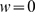.

#### Claim 3

Finally we claim that, in the limit of weak selection, for structures satisfying global updating and constant death or birth, the difference between the birth rate and death rate of an individual 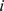 in state 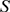 can be written in terms of the payoff of individual 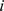 as:
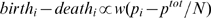
(13)where 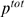 is the total payoff of players in the given state 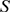.

Combining the three claims, we conclude that condition (10) is equivalent to
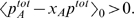
(14)Using the weighted number of interactions between players, we can rewrite the total payoffs in any given state as
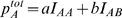


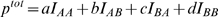
Thus, condition (14) is equivalent to
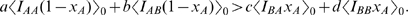
(16)However, since 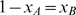, by symmetry at neutrality we have that 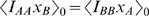 and 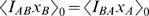. Hence (16) is equivalent to
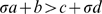
(17)where
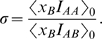
(18)This concludes the proof of the main result. Below we give the proofs for the three claims made above.

### Proofs of Claims

#### Proof of Claim 1

By assumption, either birth or death has a fixed rate; assume without loss of generality that death is constant with rate 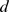. In a given state, the expected change in the frequency of 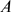 individuals is
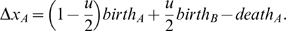
(19)We simplify this equation using the following three relations: 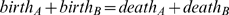 since the population size is fixed; 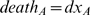 and 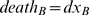 since the death rate is constant and, finally 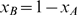. Moreover, we know that on average selection and mutation balance each other, so the average total change in the frequency of 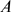 individuals is zero, i.e. 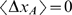. Using all these into (19) we conclude that
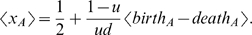
(20)This proves the claim. Note that this claim holds for any intensity of selection.

#### Proof of Claim 2

As in [41], we are assuming that the transition probabilities are differentiable functions of 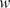 at 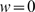. Then, in the limit of weak selection, we can write the first-order Taylor expansion of 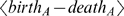 at 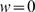


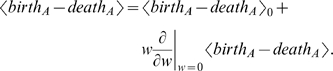
(21)For global updating, the average change due to selection in the neutral process is zero, i.e. 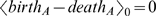. Moreover, using the product rule, we write:
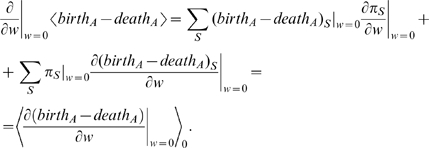
(22)Here we used the fact that for neutrality, under global updating in a fixed population size, individuals have equal birth and death rates; hence, 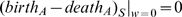 for all states 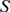. This gives the desired result.

#### Proof of Claim 3

Again, we assume without loss of generality that the death rate is constant, equal to 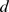. In neutrality, all individuals have effective payoff 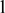. As noted in the proof of Claim 2, an individual has equal birth and death rates at neutrality, 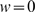. Thus, in the limit of weak selection, we can write the first-order Taylor expansion at 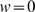 and obtain
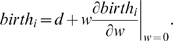
(23)When 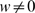, the birth rate of each individual depends on the effective payoff of any other individual, which itself is a function of 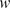: 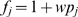. Hence (23) can be rewritten using the chain rule as
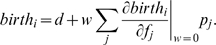
(24)Because the population size is fixed, we have 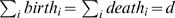. Hence, summing (24) we obtain
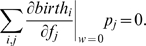
(25)When 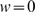 all individuals have the same fitness. Therefore, by the symmetry imposed by global updating, we have: 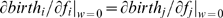 for all 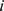 and 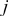 and 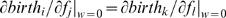 for all 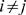 and 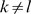. It thus follows from (25) that for each 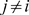


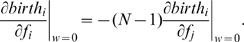
(26)Thus, we can rewrite (24) as
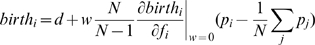
which gives the desired result.
