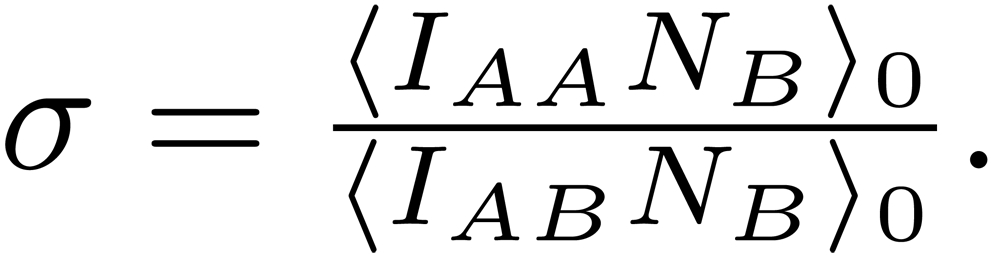# Correction: Calculating Evolutionary Dynamics in Structured Populations

**DOI:** 10.1371/annotation/064a9048-e6f7-4cf8-b259-f40cfb6696ba

**Published:** 2010-01-15

**Authors:** Charles G. Nathanson, Corina E. Tarnita, Martin A. Nowak

In the Results section, there was an error in Equation 3. Please view the correct equation here: